# Experimental Validation and Deployment of Observability Applications for Monitoring of Low-Voltage Distribution Grids

**DOI:** 10.3390/s21175770

**Published:** 2021-08-27

**Authors:** Karthikeyan Nainar, Catalin Iosif Ciontea, Kamal Shahid, Florin Iov, Rasmus Løvenstein Olsen, Christine Schäler, Hans-Peter Christian Schwefel

**Affiliations:** 1 Department of Energy Technology, Aalborg University, 9220 Aalborg, Denmark; kanr@ieee.org (K.N.); catalin_ciontea@yahoo.com (C.I.C.); fi@et.aau.dk (F.I.); 2 Department of Electronic Systems, Aalborg University, 9220 Aalborg, Denmark; rlo@es.aau.dk (R.L.O.); schwefel@griddata.eu (H.-P.C.S.); 3 GridData GmbH, 83454 Anger, Germany; schaeler@griddata.eu

**Keywords:** active distribution grids, grid observability, loss estimation, voltage quality monitoring

## Abstract

Future distribution grids will be subjected to fluctuations in voltages and power flows due to the presence of renewable sources with intermittent power generation. The advanced smart metering infrastructure (AMI) enables the distribution system operators (DSOs) to measure and analyze electrical quantities such as voltages, currents and power at each customer connection point. Various smart grid applications can make use of the AMI data either in offline or close to real-time mode to assess the grid voltage conditions and estimate losses in the lines/cables. The outputs of these applications can enable DSOs to take corrective action and make a proper plan for grid upgrades. In this paper, the process of development and deployment of applications for improving the observability of distributions grids is described, which consists of the novel deployment framework that encompasses the proposition of data collection, communication to the servers, data storage, and data visualization. This paper discussed the development of two observability applications for grid monitoring and loss calculation, their validation in a laboratory setup, and their field deployment. A representative distribution grid in Denmark is chosen for the study using an OPAL-RT real-time simulator. The results of the experimental studies show that the proposed applications have high accuracy in estimating grid voltage magnitudes and active energy losses. Further, the field deployment of the applications prove that DSOs can gain insightful information about their grids and use them for planning purposes.

## 1. Introduction

In the future distribution grids, high penetration of renewable sources such as solar and wind is expected, transforming them to be more “active” concerning power flows [1,2]. The operation and power management of active distribution grids will be challenging for the distribution system operators (DSOs) [3]. Nevertheless, the introduction of renewable energy sources (RES) provides challenges to the grid, specifically in terms of power quality. On the other hand, RES, such as photovoltaic (PV), are not only a source of grid-related measurements, but they can also provide actuation opportunities based on the measurements coming from the PV inverters. Similarly, the massive deployment of smart meters provides huge opportunities for the DSOs to obtain valuable information about the low-voltage (LV) grids.

Smart meters and PV Inverters not only provide access to additional measurements of the LV grid without the need for deployment of additional sensor hardware—they in addition provide actuation possibilities, which can be utilized for grid operation. However, this actuation needs be coordinated with other existing grid control approaches. Such control coordination approaches need to take the heterogeneity and potentially varying reliability of the different actuation devices and of the ICT connection into account and provide robustness on a system level rather than by highly reliable individual components. Therefore, leveraging the benefits from smart meter data and PV inverter data for the distribution system operation and achieving observability in the low voltage grid provides several technical challenges. These challenges include, but are not limited to [4,5]:

1. Algorithmic challenges to deal with varying data quality and with potentially inaccurate data: Data from the smart meters and PV Inverters need to be correlated to the low-voltage grid topology and processed jointly with measurements from the distribution grid from other DSO systems (e.g., substation measurements). It is pertinent to mention that these measurements might come from devices created by different vendors with varying data standards.
2. Interconnection of different systems with different criticality levels: Data access to SMs and Inverters is performed via different ICT technologies, in the latter case also involving the Internet. Therefore, the variable properties of the used communication technologies and the different connection architectures influence the security, availability, and performance of the ICT connection.
3. Security and Privacy challenges: Grid measurements close to the customer site might contain potentially privacy sensitive data, depending on the type of measurement.

The above challenges are aggravated for small- and medium-sized DSOs. In several European countries, the majority of the population is served by distribution grids that are set-up and operated by local small- or medium-sized DSOs serving up to 100,000 customers. In order to be an attractive solution for these small- and medium-sized DSOs, interoperability and customizability of the solution to work with a variety of existing deployed DSO systems is a prerequisite. Furthermore, the solution should be affordable, that is, it can run on off-the-shelf computing hardware and must be able to utilize different already deployed communication technologies to connect to remote devices or actuators. Thus, it is necessary, and a need of the hour to develop solutions that use existing communication technologies to leverage measurement functionality from smart meters and PV inverters in the LV grid. The solution should correlate this data with information from existing DSO subsystems, in order to enable and develop novel LV grid observability applications for voltage quality, grid operation efficiency, and LV grid outage diagnosis. The achieved observability can be subsequently used by specifically developed novel control coordination approaches, which utilize the existing smart meters and PV inverter actuation capabilities in conjunction with selected existing DSO actuation for voltage quality enhancement and loss minimization in the LV grid.

Developing such a solution will allow small and medium-sized DSOs the reduction of time to outage diagnosis by 70%, the reduction of grid-losses in the LV grid by 10%, and the anticipation and mitigation of 60% of the upcoming voltage quality problems for scenario of increased distributed generation without deployment of additional grid infrastructure [6,7]. As a consequence of the increased observability and novel control coordination, LV grid reinforcement investments for increased hosting of DER are targeted to be reduced by 30% in comparison to the currently used worst case-planning methods.

The energy transition heavily stresses the low-voltage grid, since a large share of the renewable generation and new load types, such as heat pumps and electric vehicle charging, are connected to the low-voltage grids [8,9]. There is therefore a strong need for grid investments [10] and to target grid investments towards the actual needs within the low-voltage grid. The detailed knowledge of the historic actual grid behavior and an accurate model of the distribution grid via a digital twin will be an essential enabler of such a targeted grid investment, and therefore is expected to reduce grid investment and grid operation costs significantly.

In conventional distribution grids, the power flow is unidirectional from the substation to customers. With the massive integration of RES, the power generation may exceed the consumption in few distribution grids which can cause reverse power flows [11]. The operation of such active distribution grids is challenging, as the node voltages and line currents are subjected to a wide range of variations and may even exceed the allowed limits [3]. It is necessary to know the system states which are generally considered to be the node voltages for corrective measures by the grid operators [12]. In addition, there is a threat to network operators from bidirectional power flow causing operational issues for network security and voltage balance [2], which forces network operators to carry out a security assessment. For this objective, network states (voltage magnitudes (V) and angles at each bus) should be evaluated under all operating conditions [3].

The majority of the DSOs do not have full information about grid power losses and the loading of grid assets, such as cables and substation transformers, at present. Typically, DSOs use a simple method to compute grid power losses in which the sum of net power consumption is subtracted from the total supplied power from the substation transformer. However, losses estimated by the above method may not be accurate due to errors in power measurements and non-technical losses [13]. Measurements from smart meters installed at customer premises can be used for calculation of grid power losses by deploying a loss calculation algorithm [14]. The information of power losses will be valuable to DSOs to find ways for minimizing the future grid power losses. Some of the measures that can be taken are reconfiguration of the grid topology, grid upgrades by which new cables and switches can be added, siting of new renewable power generations, and so forth.

Distribution system operators (DSOs) want to minimize/avoid the occurrence of under-voltages, over-voltages, dips, swells in order to provide adequate voltage margin for the integration of renewable sources [15]. The following are the most important requirements of the grid observability applications (i) to monitor voltage profiles from the measurements of smart meters, PV inverters, and electrical measurement (EM) devices connected by Remote Terminal Units (RTUs), (ii) to detect voltage variations outside a specified band, initiate alarm events to DSOs, and identify the portion of the grid experiencing voltage violations using visualization tools, (iii) to aid in the diagnosis of voltage quality problems, recommend solutions for voltage quality improvement, (iv) to estimate the active power losses in the cables using historical grid power flow data, (v) to analyze the estimated values of active power losses at different time intervals and detect any anomalies related to nontechnical losses including power theft, (vi) to minimize the grid losses, which will in turn reduce their operating expenditure and make the grid more energy efficient, and (vii) to help in the planning process, minimize investments, avoid costly grid upgrades, and for the optimum utilization of assets.

The main contributions of this paper are (i) an architecture and a reference implementation of a smart grid solution which incorporates additional layers on top of the existing systems to implement grid observability applications, (ii) build up a digital twin of the low-voltage grid based on heterogeneous data sources, (iii) a novel gateway based on the information and communication technology that acts as a brain for the whole framework and not only connects various data sources but also responsible for coordination of observability applications, (iv) a definition and realization of two-grid monitoring applications, namely, grid monitoring and loss calculation, (v) an assessment and validation approach of the accuracy of the digital twin and of the two observability applications, and (vi) validation and assessment of the observability applications in a laboratory setup.

The organization of the paper is as follows. Section 2 presents the proposed framework for deployment of grid observability applications. The individual layers, their interconnections, and their interfaces to the distribution grid are explained. In Section 3, an overview of the two observability applications (grid monitoring and loss calculation) is provided, followed by Section 4, in which the laboratory experimental setup using real-time hardware in the loop (RT-HIL) for validation of the two applications are explained. In Section 5, the results obtained from the RT-HIL studies are presented, and the results from field trials of the applications are given in Section 6. A short summary, discussion, and conclusion about the deployment studies of the observability studies are provided in Section 7.

## 2. Framework for Grid Observability Apps Deployment

### 2.1. Overview of Information Flow

Manufacturers usually equip PV inverters with on-board monitoring systems which suit the needs of the (residential) plant operator. For PV systems connected to the LV level (i.e., for residential PV), data logged and cached by the inverter (e.g., power flows, AC voltages, etc.) are commonly sent to an online platform, which provides monitoring access for the plant operator. For non-residential PV plants (i.e., for commercial installations), third-party monitoring equipment is widespread, which also integrates communication paths for monitoring and remote control by the DSO or by a trading agent or aggregator. For LV-connected generators, an information flow of inverter data to the DSO, for whatever operational purpose, cannot be considered as state-of-the-art.

The PV inverter as a non-DSO source of potentially DSO-relevant data on a LV level should be made accessible. One key advantage of this approach is the fact that the inverter is an existing source of data and therefore is expected to be exploitable for the DSO at relatively low additional cost (compared to the cost for a monitoring system set up for DSO-purpose only). Besides analysing the availability and consistency of this new data source, interfaces should be specified (covering aspects of secure server-to-server communication), tested, and implemented to be employed by the grid observability applications.

Furthermore, with the ambition of reaching 70% replacement of electricity meters with smart meter roll-out by 2020 across EU [16], there is huge potential for increasing the grid observability of the low-voltage distribution network. Until now, the main focus for the smart meter has been to provide information about consumption and/or production for consumers/prosumers and other market actors [17,18,19]. The utilisation of the smart meter as a sensor on the low voltage grid has had less attention, although it is anticipated that it can, in combination with other smart grid devices, provide competitive advantages for the DSOs in order to realise a more cost-efficient operation [19].

Employing the available measurements from PV inverters and smart meters in addition to available data from DSO-based systems, such as grid topology information, suitable estimation algorithms need to be assessed and implemented for the distribution grid in order to support grid observability applications [4,5,20,21]. These observability applications should be able to run on top of a gateway and provide visualization interfaces to the DSO staff via a graphical user interface (GUI), as well as necessary information for the control and coordination algorithms. In the proceeding subsections, an architecture is presented that fuses information from different data sources (such as AMI, PV inverters, grid topology information etc.) to assist deploying grid observability applications.

### 2.2. Grid Observability Applications Deployment Architecture

In the context of deploying observability applications in LV distribution grids, the Reference [22] presents a decentralized monitoring solution to achieve real-time LV network monitoring utilizing smart meters and secondary substation measurement devices to gain accurate information of the LV network. It also uses the device on the secondary substation to aggregate and analyze data first before communicating to the control center. This solution, however, is neither modular nor does it use the measurements for improving the power quality, as well as the outage management. Based on the Romanian 440 kV and 220 kV power transmission networks, the Reference [23] develops network design problems to address the security and resilience requirements, as well as the provisioning of cost-effective smart grid communications. The security requirement is formulated in terms of the monitoring of communications by smart grid intrusion detection systems, whereas the resilience requirement addresses the ability of the infrastructure to function in the presence of, for example, failure or cyber-attacks. However, although this solution is scalable and helps in real-time monitoring of the LV grid, it does not assist in addressing the power quality issues and outage management.

The Reference [24] proposes a hierarchical transactive control architecture that combines market transactions at the higher levels with inter-area and unit-level control at the lower levels. This multi-level architecture operates over time-scales that range from seconds to minutes. As with [25,26], the authors in [24] mainly focus on the scalability and modularity of their proposed architecture, but they do not address how this architecture can be used to deploy LV grid observability applications.

**Figure 1:**
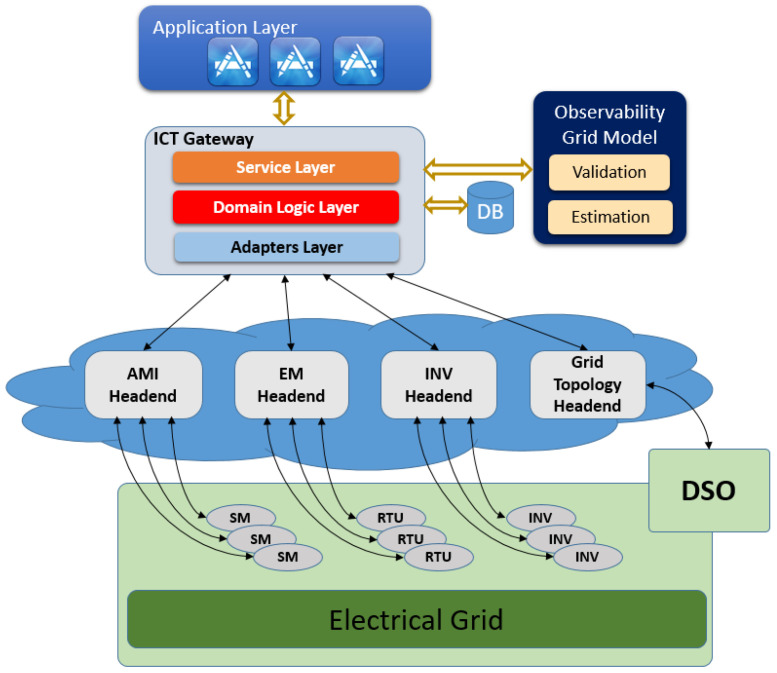
High-level system architecture for deployment of smart grid applications.

This paper presents a framework that can be used for deploying the grid observability applications (such as LV grid monitoring, loss calculation, outage detection, and diagnosis, etc.), as well as control applications. The novelty of this framework lies in the introduction and development of a central component called the ICT gateway which acts as the brain of the whole framework (for details of ICT gateway, see Section 2.2.2). So far, DSOs have several systems where data are stored, like geographic information systems, or which collect data, like Smart Meters. Since these systems are heterogeneous, linking data from all these systems requires much manual work or—if they have the IT expertise—implementation effort from the DSO side. In contrast, the ICT Gateway connects to all these heterogeneous data sources, brings the data from these sources together in a harmonized form, and links them. As a result, the DSO has harmonized data without any effort on their part, or the need to buy new hardware. The high-level system architecture for deployment of smart grid applications is shown in Figure 1. This architecture shows four main layers that we explain below: the Application Layer, the ICT Gateway (along with a Database), the head-end layer, and the Electrical Grid [27].

#### 2.2.1. Application Layer

The Application Layer includes novel applications aiming at supporting the DSO in three main areas: Operation Efficiency, Voltage Quality, as well as Outage Detection and Diagnosis. Each of the applications run on top of the ICT Gateway and retrieves the grid topology information, the measurements, and the events through the ICT Gateway. A description of the main applications is given in Section 3.

#### 2.2.2. ICT Gateway Layer

The ICT Gateway is in charge of establishing connections to the different measurement and topology data subsystems. Due to heterogeneity of subsystems deployed in the field, the ICT Gateway needs to adapt the collected data in a uniform format to be easily stored in the database and provided to the GUI and applications that will request them. Furthermore, the ICT Gateway is responsible for mapping the measurements coming from different subsystems to the specific nodes in the grid topology they belong to. This mapping is in itself a huge challenge because the measurements in the real field usually belong to different subsystems/devices following different standards. The task of the ICT Gateway is therefore divided into multiple layers, as illustrated in Figure 1. The ICT Gateway has a classical three-layer architecture consisting of the service, domain logic, and adapters layer.

**Service layer:** The service layer provides a REST API used by the applications and GUI in order to retrieve data from the database or trigger applications.

**Domain logic layer:** The domain logic layer consists of modules for the connection to the database and the needed algorithms for data processing. Particularly relevant are the measurement and topology module. Each of these two modules has to perform a challenging task: The first one is responsible for pre-processing measurements, such as respective topology and data from different and heterogeneous subsystems, and storing it in the database. The second is responsible for querying and postprocessing (e.g., unit conversion) the respective measurement or topology data from the database.

**Adapters layer:** The adapters layer provides interfaces to the different subsystem headends. An adapter processes data retrieved from a headend, and forwards it to topology and measurement modules in the domain logic layer. Specifically, it includes adapters for the AMI subsystem, the inverter subsystem, the EM subsystem, as well as for the grid topology data.

1. The AMI adapter refers to the Advanced Metering Infrastructure (AMI) subsystem aiming at collecting and making available to the ICT Gateway data from SMs deployed in the grid.
2. The INV adapter refers to an internet portal of the PV inverter subsystem. It aims at three tasks: (1) Collecting and making available data from PV systems deployed in the grid, (2) providing service messages that represent alarms generated within the PV system, and (3) sending data like setpoints to PV inverters.
3. The EM adapter refers to the Remote Terminal Unit (RTU) subsystem. It aims at collecting measurements and alarms from electrical measurement devices deployed in the grid, and sending on-demand access requests to the RTUs.
4. The Grid Topology adapter refers to the GIS system of the DSO for collecting electrical grid topology information, like nodes and cables, as well es their parameters.

**Observability Grid Model:** The data required by applications may not always be available, as it will be too costly to equip every potentially interesting grid location with a measurement device. Even for grid locations with measurement devices, data may be temporarily unavailable due to communication delays, loss of data during transmission, or failure of measurement devices. The purpose of observability grid model (OGM) is to use the available data and the grid mathematical model to estimate the values of the missing data. The OGM can be triggered by any application which needs the missing measurement data for a specified time duration.

#### 2.2.3. Headend Layer

The headends are used to connect different physical data sources such as AMI, the PV inverters internet portal, RTUs and the GIS system with the adapters layer of the ICT Gateway. For each DSO, one usually needs one headend for the topology, and one for each type of measurement device. Typically, the topology headends differ from DSO to DSO, particularly due to a lack of standardized GIS system data storage schemes and export functionalities.

### 2.3. Grid Topology Extraction

In order to achieve observability of LV grids, the data from the SMs, Inverters, and other measurement devices of the DSO needs to be correlated to the LV grid topology. Mostly, the observability applications are activated by a user command through the GUI. These applications should be executed based on the grid topology and measurement data; therefore, these should get the grid topology and subscribe to topology changes. The gateway must maintain a representation of the MV and LV grid topology and be able to expose it to the applications that have subscribed to them. A subsystem should be developed that should provide access to information about the distribution grid topology and the connected consumers, generators, and prosumers. This subsystem should be accessed by the gateway via a grid topology headend.

However, extracting grid topology information and fusing the heterogeneous measurements’ data at DSO provides multiple challenges, such as [5,18]:

1. Lack of standard data models: DSOs frequently include the LV grid topology data in their databases, which may come from different systems such as Geographical Information System (GIS) or other asset management systems, which store a relevant part of the grid topology in a type-specific format. For some DSOs, this data may come in a single file (e.g., in a common information model (CIM) XML format), while for others, it may come in different files, where each file carries information about a specific grid node entity. The varying data quality of grid topological information is due to the lack of standard data models that leads to complexities in extracting topology information.
2. Complexities in extracting topology information: The topology data usually employ a composite design pattern, and thus, entities cannot be directly interlinked, that is, there is no explicit mapping between a substation and a customer connection box, and so forth. Therefore, novel algorithms are required for not only extracting and parsing the topological data, but also to create links between different entities of the grid.
3. Dependence on multiple databases: Information about different grid node entities need to be collected from different databases. For instance, the GIS system usually stops at the house connection box. Thus, the private cables from the connection boxes to the customer meters are not included in the GIS data and need to be collected from other database sources.
4. Incorrectness/incompleteness of topology information: The inaccuracies/deficiencies in the data can be addressed by either manually defined rules in implementation or depending on multiple databases to extract correct information within a required amount of time [18].
5. Mapping between data and measurement points in grid topology: Data from devices such as RTUs, smart meters or PV inverters need to be mapped to the measurement points in the grid topology. Typically, several layers of mappings exist because each subsystem uses its own internal mappings, while DSOs have their own schemes. This makes it even harder for systems to link data properly [28].
6. Parameter fetching issues: Various grid parameters, specifically cable parameters, might get outdated or need to be changed, which are often fetched from datasheets. Manual fetching of such parameters becomes challenging for systems.
7. Challenges in data collection and integration: Collection of measurements from various devices (PV inverters, RTUs) with diverse identification schemes is a challenge. The measurement data from devices need to be related to a unique grid topology model.

#### Observability Grid Model

**Figure 2:**
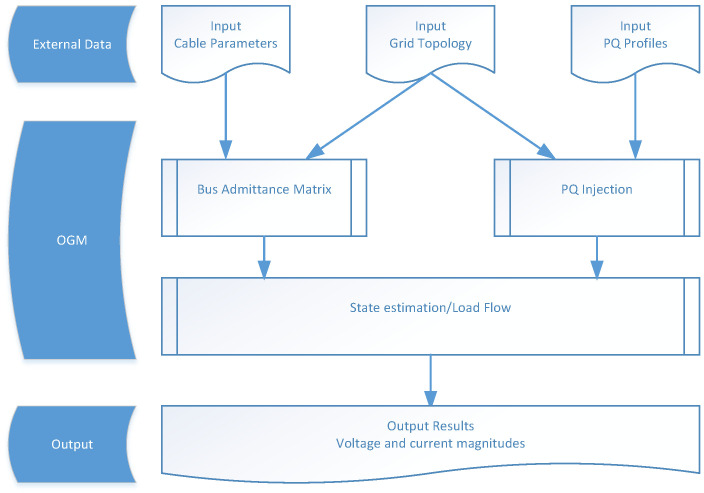
Proposed structure of OGM [29].

The observability grid model (OGM) implements a load flow algorithm [29].It is used to calculate electrical parameters for the grid observability applications (e.g., calculate voltage values for all grid nodes in the LV topology for grid monitoring), as well as to estimate and complement missing data (cable lengths and types, loads, etc.). The structure of the OGM is shown in Figure 2. The inputs to the OGM are the grid cable parameters, grid topology, and the 15 min average active and reactive power consumption and generation at each customer connection point. From the cable parameters and grid topology, the bus admittance matrix is calculated. Using the grid topology and the active and reactive power (PQ) profiles, the PQ injections (the net active and reactive power consumption) at each node are calculated. The OGM employs either a load flow algorithm or an advanced state estimation algorithm to compute the voltage and current phasors at each node.

Applications can trigger the OGM over the ICT Gateway for a specific time interval $\left[t_{1},t_{2}\right]$. Upon this trigger, for every time stamp $t\in [t_{1},t_{2}]$, the ICT Gateway sends the following inputs to the OGM:

1. The bus admittance matrix is obtained from the distribution grid topology and the cable parameters (series resistance and reactance and shunt capacitance);
2. Measured voltage values *V* at time *t* of the transformer (lumped value over phases); and
3. Measured active *P* and reactive *Q* power values at time *t* of all customer connection boxes (sum over phases).

The algorithm to compute the node voltages and line current phasors has to be properly chosen based on the accuracy requirements, computational burden, and applications that use them. There are many methods available in the literature, such as load flow programs based on the Newton–Raphson (NR) method, and state estimation algorithms based on the nonlinear weighted least squares method or extended Kalman filter [30]. If the grid is highly unbalanced, individual phase voltages and currents have to be estimated using a three-phase load flow or three-phase state-estimation algorithm [31]. In this work, the NR method-based load flow algorithm is employed in the OGM block. Utilization of advanced state estimation algorithms will be a part of our future work.

Based on this input, OGM calculates the following operational parameters: (i) Voltage values of all grid nodes (lumped value over phases), and (ii) current values of all cables (lumped value over phases).

With this description of the framework, it is clear that the framework proposed in this paper is different from the Sunspec modus [32], which is an open communication standard that specifies common parameters and settings for only monitoring and controlling the DER systems.

### 2.4. GUI as a Visualization Tool

**Figure 3:**
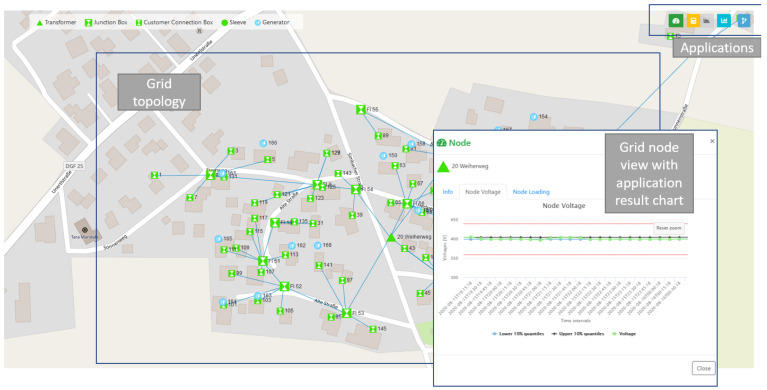
The GUI of the proposed system.

GUI allows the DSO operators to interact with the system by providing an input and output for both the Applications and ICT Gateway functionalities. It is designed as a web application the DSO can access over a web browser allowing the DSO to use the system from various clients (PC, tablet etc.) and without installing additional software. As illustrated in Figure 3, in the GUI, the DSO can see the LV grid topology and trigger applications. By clicking on nodes and cables, he or she can view their parameters and results of ran applications. As Figure 3 illustrates, application results are visualized with charts in order to provide intuitively comprehensible information to the DSO. The following section (Section 3) provides an overview of the observability applications selected for this paper.

## 3. Overview of Grid Observability Applications

The main advantage for the DSOs to adopt the proposed solution shown in Figure 1 will be the deployment of smart grid applications that facilitate cost-efficient and reliable infrastructure operation of distribution grids. The applications can enable the DSOs to draw benefits from smart meter measurements, but also PV inverter measurements, and hence create additional return on the deployment investments. These applications have been selected based on the user stories provided by medium-sized DSOs in Denmark and Germany, thus having a high and concrete demand from other DSOs as well. In this paper, the following two observability applications, namely, Grid Monitoring and Loss Calculation, are studied, and the challenges in their deployment are analyzed. Other applications, such as outage detection, outage management, grid planning and automatic voltage regulation will be part of our future work. The following subsections provide details of the two selected applications.

### 3.1. Grid Monitoring Application

The Grid Monitoring (GMon) application allows the DSO to specify a time interval $\left[t_{1},t_{2}\right]$ in the past, and then uses average voltage values (for, e.g., 15 min average values) in order to provide the following output for each grid node through the GUI:

1. The average voltages over the full time interval $\left[t_{1},t_{2}\right]$;
2. The time stamps $t\in [t_{1},t_{2}]$ where the average voltages exceed the configurable threshold; and
3. The evolution over $\left[t_{1},t_{2}\right]$ of the 95% and 5% quantiles of the average voltages over a one-week sliding window [15].

GMon can either use measured average voltages, or voltages calculated with the OGM. The output of GMon contains valuable information about grid voltage quality, including time.

### 3.2. Loss Calculation

The Loss Calculation (LC) application allows the DSO to specify a time interval $\left[t_{1},t_{2}\right]$ in the past, and then uses the measured average active and reactive energy values to calculate the power losses. Similar to GMon, the LC application operates on the historic data, and losses are computed at least 24 h back from the moment of execution of the LC application. The grid topology is assumed to be static during the full time-interval for which LC computes the losses. A lumped value for three-phase powers are provided to the LC application—that is, the average active and reactive powers are available as each one three-phase value for each direction and for each 15 min interval for all grid nodes, either by smart meter measurements or by calculations from the OGM. The outputs of the LC for each time stamp $t\in [t_{1},t_{2}]$ are the following.

1. Active and reactive powers at the substation transformer;
2. Absolute and relative, active and reactive power total loss in the grid.

The results of the Loss Calculation will be helpful for DSOs to analyze if there is an unreasonable amount of non-technical losses which may be due to errors in the metering system, communication errors, or theft of power. DSOs can further carry out an investigation to find the reason behind such unaccountable power losses and fix them. Alternatively, the power losses could be purely technical losses, that is, ohmic losses in the cables. In that case, DSOs can consider solutions which include grid reconfiguration, volt/var optimization, and grid upgrades to improve the grid efficiency.

## 4. Experimental Setup for Validation

### 4.1. Description of RT-HIL in the Context of App Validation

**Figure 4:**
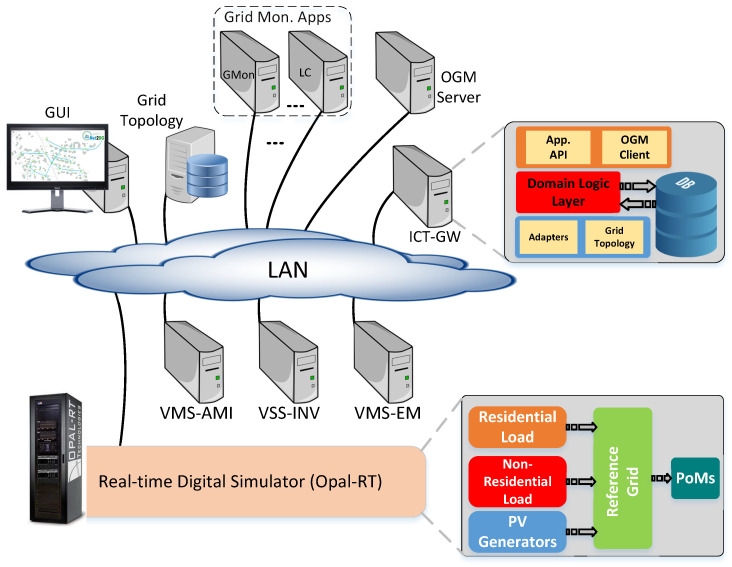
Laboratory RT-HIL setup for testing and validation of observability applications.

The smart grid applications and the entire ICT structure discussed in this paper are tested and validated in the Smart Energy Systems Laboratory of Aalborg University, which was specially built for such work [33,34]. This system is centred on a Real-Time Digital Simulator where distribution grids, customers, and the measurement points are captured in a configurable model. Several models and tools have been developed and tested to allow flexible integration of ICT gateways in a realistic, close-to-real-life way [33]. For this work, the RT-HIL setup shown in Figure 4 is used, which consists of the following components: an Opal-RT digital simulator, a high-speed Ethernet/LAN switch, and ICT hardware equipment, that is, computers and servers. The ICT hardware is used to host the ICT gateway, a virtual measurement system (VMS) for acquiring smart meter measurements, a virtual subsystem (VSS) for acquiring PV inverter measurements, and last but not least, the observability applications.

At the heart of the RT-HIL setup, there is an Ethernet switch which ensures connection between all the related entities. This switch has been configured to provide the necessary network setup and enables the success of various components to each other. Therefore, the Ethernet/LAN switch interconnects all ICT hardware (including the ICT gateway, GUI PC, and PCs hosting VMS-AMI, VSS-INV, grid topology, grid monitoring applications, etc.) and the Opal-RT digital simulator [33].

The Opal-RT digital simulator, based on multi-CPU hardware, is capable of real-time simulation of extensive electrical grids, electrical equipment, and other assets of such grids. The simulated grid, equipment, and assets are modelled in RT-LAB, the proprietary software of Opal-RT, which is compatible with Matlab/Simulink. In practice, this is very convenient, as the Simulink models of various electrical equipment can be directly used in RT-LAB for Opal-RT-based simulations. In this study, the Opal-RT digital simulator is used to emulate an LV distribution grid, including its metering and control equipment.

The Opal-RT allows not only the emulation of an electric network and its associated equipment, but also manipulation of the electric network, including its voltage and current profiles, network topology, and other relevant parameters in order to generate relevant events for the testing and validation of the ICT applications. In other words, the electrical network, its loads and generators, and all electrical parameters can be adjusted in such a way that the desired type of network event is simulated. These can be under-voltages, over-voltages, short-circuits, normal operation conditions, and more. As such, the ICT applications can be tested and validated for different types of network events. More details regarding the structure and operation of the Opal-RT and laboratory RT-HIL setup in which it is used can be found in [33,34].

The distribution grid model is basically a digital twin of a real distribution grid in Denmark, though modified accordingly to support various operational scenarios, for example, increased penetration of solar PV, and outages in selected locations (see Section 4.3). The AMI infrastructure from the smart meter to the head-end system uses virtual models for smart meters, the data collection mechanism, and the headend system, including databases called the VMS-AMI. A similar approach is used to capture the Solar PV inverter system and the head-end server that connects to RTUs named the VSS-INV. In particular, the Solar PV emulator captures the bidirectional data flow from the Inverter to the control applications (such as Automatic Voltage Regulation (AVR)) via the headend and ICT gateway and back to a specific inverter according to generated references by AVR. A dedicated platform is used to emulate specific communication networks and their data traffic characteristics using trace-based measurements from real systems.

As discussed in Section 2.2, the ICT Gateway collects grid topology information from Opal-RT via a grid topology headend, and it receives smart meter measurements through the VMS and maps these information such that each measurement is associated to the grid location it is coming from. This mapping is essential for the grid monitoring applications to perform several calculations.

### 4.2. Summary of Hardware/Software Platforms

Given a variety of hardware/software tools used in this work, Table 1 summarizes the involved suites, indicating the tools and technologies used to develop those software.

### 4.3. Description of the LV Distribution Network

**Figure 5:**
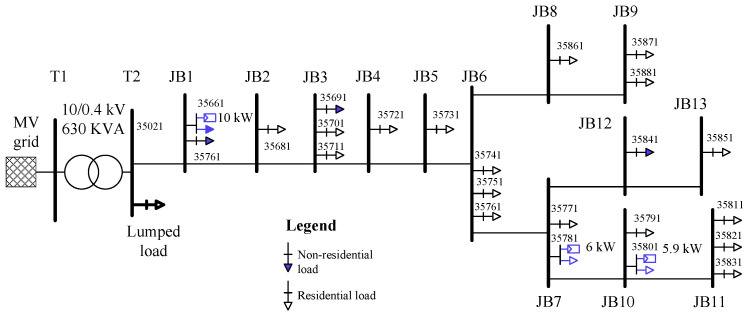
Schematic diagram of a representative LV distribution grid in Denmark used for the study.

Figure 5 presents the single-line diagram of the LV distribution grid used for testing and validation of the discussed applications. It consists of 23 three-phase residential consumers supplied from a MV/LV power transformers via 13 distribution boxes labeled JB1, JB2, and so on. Each distribution box provides electricity for up to three consumers. The smart meter ID at each customer connection point is denoted by a five-digit number, for the measurement device connected to the secondary side of LV transformer that is 35021. The electric parameters of the constituent equipment of the LV distribution network are given in [35]. Three out of the 23 consumers of the LV network possess a three-phase PV system that can generate electricity and which, if it exceeds the local consumption, will be injected into the grid. Accordingly, those consumers would become electricity producers. The installed power and location of the PV systems are shown in Figure 5. It should be noted that each PV system is an in-house unit, connected to the main grid through the same smart meter as its associated residential consumer. The smart meters, one for each consumer, are not shown in Figure 5.

The described LV distribution grid is modeled and emulated using the Opal-RT digital simulator. An equivalent PI-model is used for the electrical cables, an equivalent Thevenin model is used for the MV grid and MV/LV transformer, and a P-Q load model with individual P and Q on each phase is used for loads and generators. The apparent power of every consumer is dictated by its own consumption profile, with each profile being obtained by measurements from the residential consumers of a real LV network [35]. Similarly, the power generated by every PV system is dictated by solar irradiance and ambient temperature profiles, which are also obtained by measurements and used as inputs for the PV models. However, unlike the load models, the PV system models can offer, if necessary, some power control options, such as the Q(V), P(V), constant power factor, and others. The consumption, irradiance, and temperature profiles are used as inputs for the Opal-RT-based model of the LV network.

### 4.4. Use Cases

For the purpose of this study, the voltages of the LV distribution grid are manipulated to obtain different sets of grid operating conditions. This is realized by varying the voltage of the MV grid shown in Figure 5, and hence generating the following:

1. Normal conditions, for which the grid voltages at all smart meters of the LV distribution grid are within the normal limits, that is, the 0.9 p.u. to 1.1 p.u. interval;
2. Under-voltage conditions, for which the grid voltages at some smart meters fall below the 0.9 p.u. limit; and
3. Over-voltage conditions, for which the grid voltages at some smart meters exceed the 1.1 p.u. limit.

**Figure 6:**
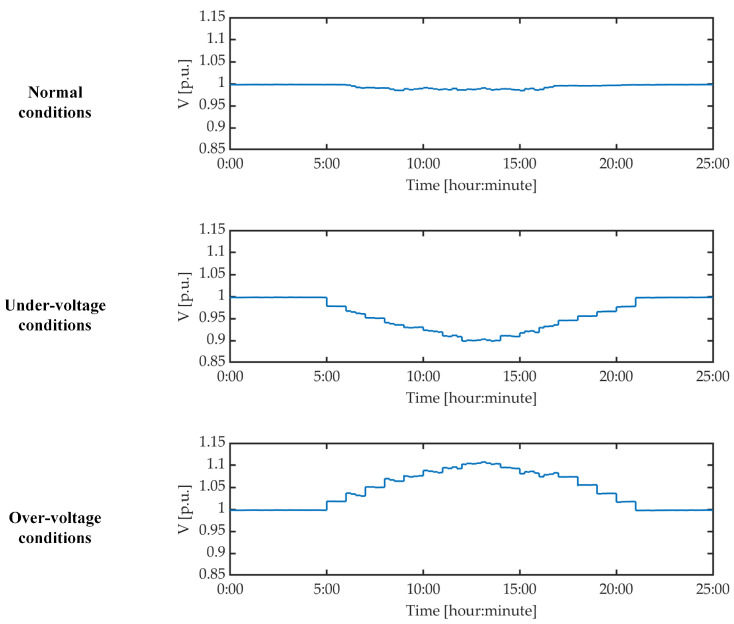
Voltage on LV side of MV/LV trafo for normal, under-voltage, and over-voltage conditions.

Figure 6 shows the grid voltage on the LV windings of the MV/LV transformer for each set of grid conditions, with a simulation duration of 25 h for each case. The transformer voltage has the same magnitude on all of its phases, so Figure 6 illustrates the voltage for only one phase of this transformer.

## 5. OPAL-RT Lab Validation of GMon Application

In this section, the observability application GMon is evaluated. First, its correctness is validated and secondly, as the performance of GMon highly depends on the OGM, an assessment of the accuracy of OGM under correct measurement and topology data are carried out.

### 5.1. Grid Monitoring Application—Correctness

In this section, the correctness of GMon without OGM is assessed. The methodology is presented first, followed by the results.

#### 5.1.1. Methodology

**Figure 7:**
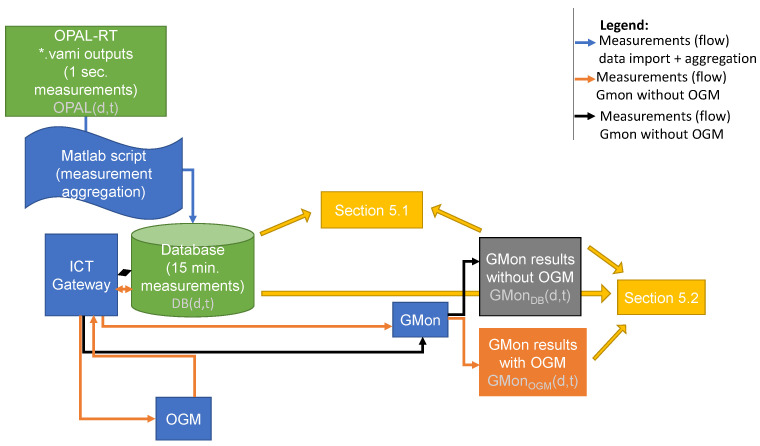
Illustration of data flow and performed comparison in the lab validation.

The overall setup of the validation is illustrated in Figure 7. The correctness of the GMon application and the APIs of the ICT Gateway used by GMon is validated using the data from the OPAL-RT lab setup in the under-voltage scenario. The respective measurements from this scenario are shown in Figure 6. An upper violation limit of 420 V, and a lower limit of 380 V was used. Consequently, under-voltage events are expected. With OPAL(d,t), we denote the 1 s voltage measurement of measurement device *d* at time *t* in the OPAL-RT trace. We averaged the measurements of the OPAL-RT lab setup outputs to 15 min measurements (to represent real-world smart meter behavior), and imported them in the database of the ICT Gateway. With DB(d,t), we denote the voltage measurement of device *d* for the interval ending at time instant *t* in the database. With ${\mathrm{GMon}}_{\mathrm{DB}}\left(d,t\right)$, we denote the GMon output of the node connected to the measurement device *d* for the same time interval ending at *t*. To validate the correctness of GMon, we compared the voltage output of GMon with the voltage values in the database for every node with one point of measurement device associated by calculating the relative error:

$$MRE_{d}=\frac{1}{T}\sum_{\mathrm{timestamp}\;t}\frac{|\mathrm{DB}\left(d,t\right)-{\mathrm{GMon}}_{\mathrm{DB}}\left(d,t\right)|}{\mathrm{DB}(d,t)}.$$

where *T* is the considered time interval. In addition, the number of voltage violation events is compared in order to evaluate the influence of a potential error on the voltage violation detection. GMon is correct if the MRE and difference in number of detected events is near zero. A small error is allowed due to rounding deviations in the ICT architecture.

#### 5.1.2. Results

**Figure 8:**
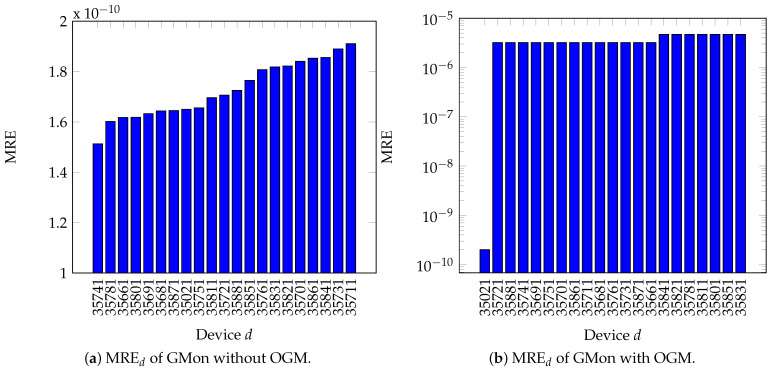
Mean relative error (MRE) of voltage values, ordered in descending order by device.

**Figure 9:**
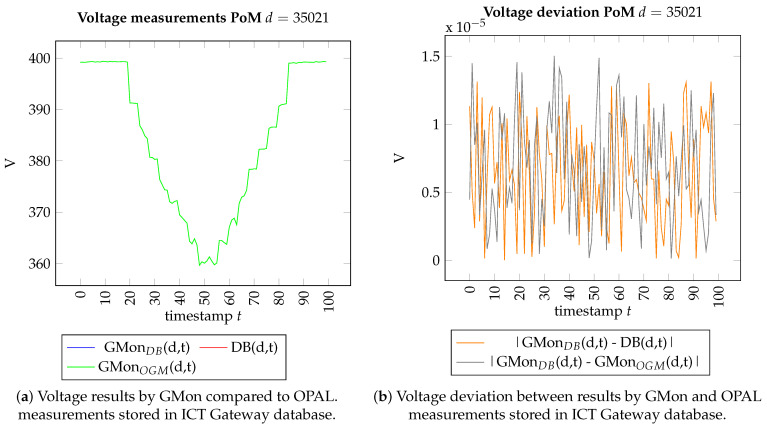
Voltages at the substation in the under-voltage scenario.

**Figure 10:**
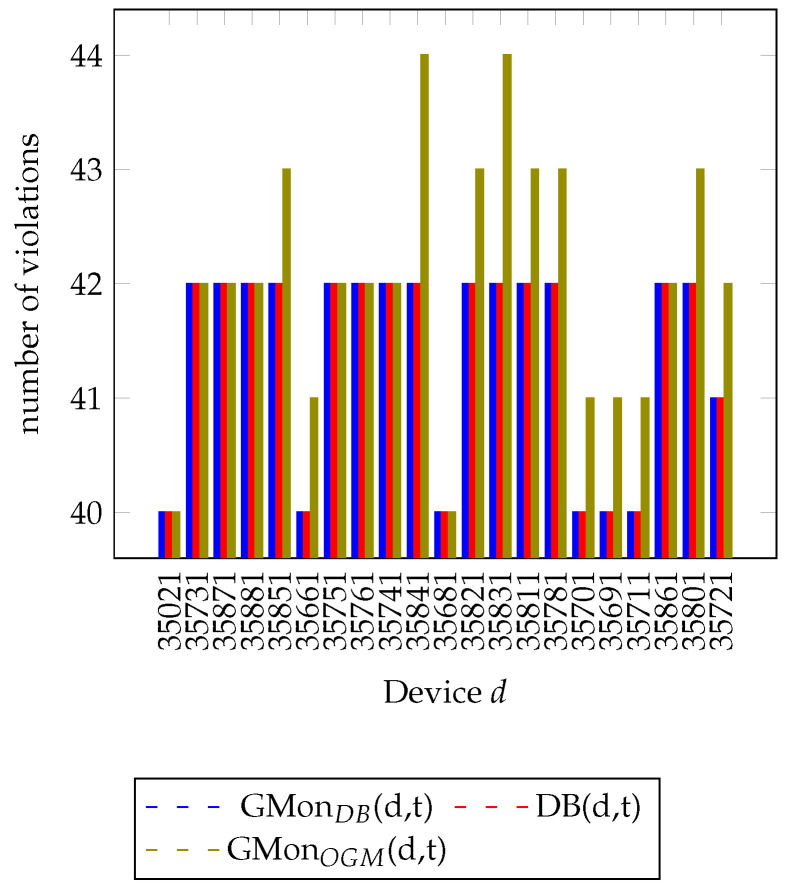
Comparison of number of undervoltage events.

Figure 8a shows the results of the correctness experiment. The MRE are smaller than $10^{-9}$ for all devices. This means that, apart from numerical rounding errors, GMon outputs the correct voltage values. Figure 9 presents the error of one device, namely, device d=35021 which belongs to the transformer in the substation, in detail. In Figure 9a, which shows the voltage measurements GMon${}_{D}B$(d,t) and DB(d,t) for all time stamps *t*, the lines are located directly on top of each other. This means that visually, there is not any error. Figure 9b confirms that the difference between both lines is smaller than a $2\times 10^{-5}$ volt and is thus not visible in Figure 9a. Due to this small error, all events are detected correctly by GMon, as shown in Figure 10.

### 5.2. Grid Monitoring Application Using OGM—Correctness

Since GMon uses OGM-calculated voltages if no measurements are available, a validation of the OGM was performed. The goal of this study was to assess the accuracy of OGM-calculated voltages. They should differ from the measured ones as little as possible. In this section, the methodology and the results are presented.

#### 5.2.1. Methodology

We validate the level of accuracy by comparing the voltages from GMon without OGM with the voltage values from GMon with OGM. With ${\mathrm{GMon}}_{\mathrm{OGM}}\left(d,t\right)$, we denote the GMon output using the OGM of the node connected to the point of measurement device *d* at time *t*. We use the mean relative error to measure the error in between:

$$MRE_{d}=\frac{1}{T}\sum_{\mathrm{timestamp}t}\frac{|{\mathrm{GMon}}_{\mathrm{DB}}\left(d,t\right)-{\mathrm{GMon}}_{\mathrm{OGM}}\left(d,t\right)|}{{\mathrm{GMon}}_{\mathrm{DB}}\left(d,t\right)}.$$

Additionally, we compare the number of detected events.

#### 5.2.2. Results

Figure 8b shows the overall results of the correctness experiment. We first observe that the MRE of device d=35021 has the smallest MRE. This is expected, as the voltage of this point of measurement serves as an input into the OGM. Figure 9 confirms that the error for this device is in the same order of magnitude as in the experiment in Section 5.1. Secondly, for all devices, the MRE is smaller than $10^{-5}$, and thus very low. Nevertheless, Figure 10 shows that these errors lead to a difference in the number of detected events. This happens if the measurement the event belongs to is near the violation border. The highest difference in the number of detected events for one device over the whole time period is two events. However, the fraction of incorrect detected events over all measurement devices and time stamps is $\frac{14}{869}<2\%$. Overall, this study proves that the accuracy of the OGM is very high.

### 5.3. Summary

The results from the lab valuation of GMon provides two key insights. First, the results prove that GMon using measured voltage values is—apart from small errors due to rounding in the magnitude of an MRE of $10^{-9.7}$—highly accurate. All events are correctly detected. Secondly, the error introduced by OGM is $10^{-5}$ and also quite low, but leads to 2% deviation in the number of detected events. Thus, in total, we see that GMon is correct, and due to high OGM accuracy, valuable for the DSO also in only partially measurement scenarios.

### 5.4. Loss Calculation Application Using OGM—Correctness

Similar to the validation of the GMon application, the loss calculation application also depends on OGM for the calculation of grid power losses. The goal of this study was to assess the accuracy of energy losses calculated by the LC application using OGM for a period of 24 h with a sample time of 15 min. The energy losses calculated from the 15 min average values of OPAL-RT data are used as the true values for comparison of results.

### 5.5. Results

**Figure 11:**
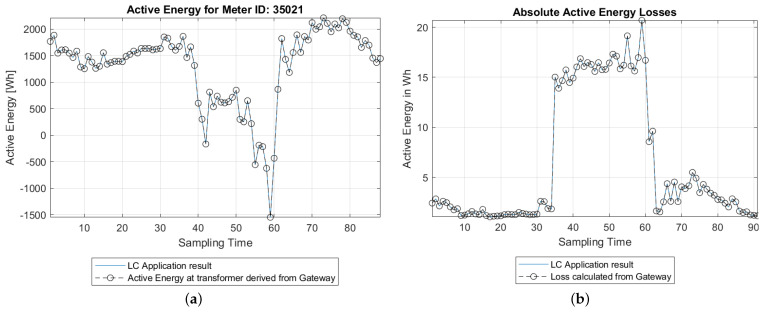
Plot of the (**a**) Active energy at node ID 35021 during two consecutive days; (**b**) active energy losses at TME over two consecutive days.

Figure 11 shows the results from the LC application compared with that of the OPAL-RT data. Figure 11a shows the active energy supplied by the substation transformer and Figure 11b shows the absolute total energy losses in the distribution grid under study. From the above plots, it can be seen that the estimation energy matches closely with the true values at all time periods. A summary of the results is provided in Table 2. From the results, it is clear that the LC application is highly accurate in estimating the energy losses. DSOs can make use of the LC application to know the historical energy losses in their distribution grids. These values will be useful in the planning of grid upgrades.

## 6. Field Trials of Observability Applications

The proposed grid monitoring system is deployed in two different field trials of a German and a Danish DSO. These trials demonstrate the successful deployment of the whole system for DSOs. In the following, due to a full smart meter roll-out in the Danish setting, preliminary insights of the Danish field trial regarding the grid monitoring and loss calculation application are provided.

### 6.1. Description of the Danish Field Trial

The Danish field trial is located in northwestern Denmark. There is a secondary substation connected to five low voltage feeders, and 98 customers (household, small industry, public institutions, supermarket, etc.) partially equipped with solar PV installations. There is 100% roll-out of Kamstrup-smart meters in the area. Headends for topology [17], substation transformers, as well as Smart Meter measurements are implemented in our deployment. The grid used in the validation (see Section 4.3) equals one of the feeders of the field trial.

### 6.2. Grid Monitoring Application

**Figure 12:**
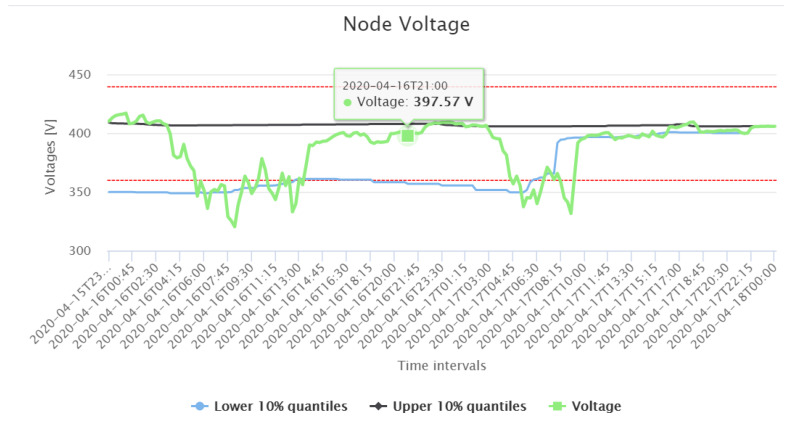
Under voltage situation near the 3011 substation. Red: thresholds for overvoltage and undervoltage.

The primary goal of this application is to detect voltage violations in the grid. Generally, the grid of our Danish DSO is fairly robust. To simulate a scenario violating the voltage band, the electrical parameters of a cable connecting an industrial consumer are manipulated in the digital grid model, replacing it by a significantly thinner cable. Then, GMon with OGM calculates the respective voltage at all nodes. The result is illustrated in Figure 12: The GMon graph of our Danish DSO shows clearly that the resulting voltages are beyond the threshold. In the GUI map, the respective node is also highlighted in red. By this demonstration, it is shown that the DSO is able to recognize issues in grid topology, leading to voltage violations.

### 6.3. Loss Calculation

**Figure 13:**
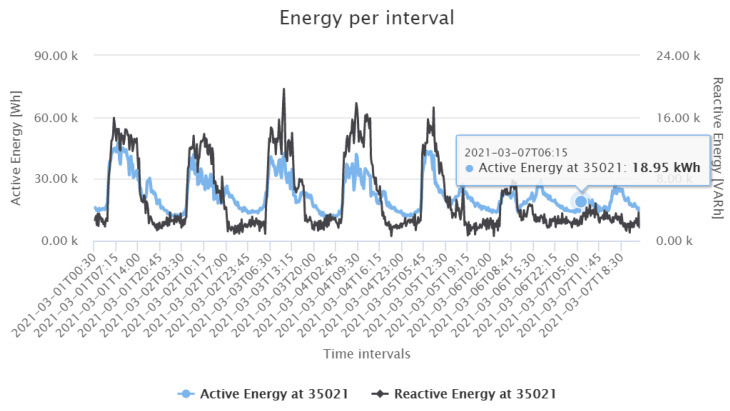
Active and reactive energy at device 35021 during the first week of March 2021.

**Figure 14:**
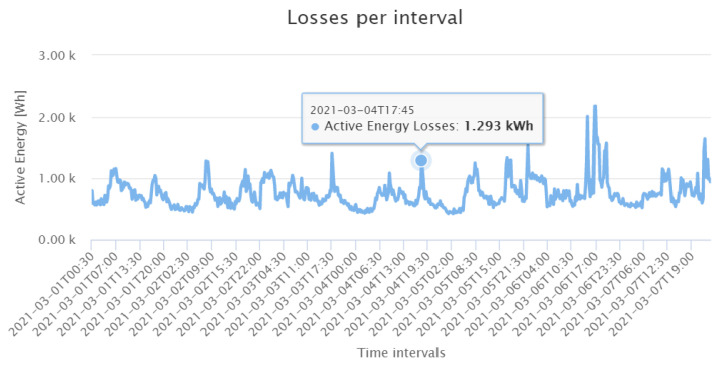
Active energy losses occurred during the first week of March 2021.

**Figure 15:**
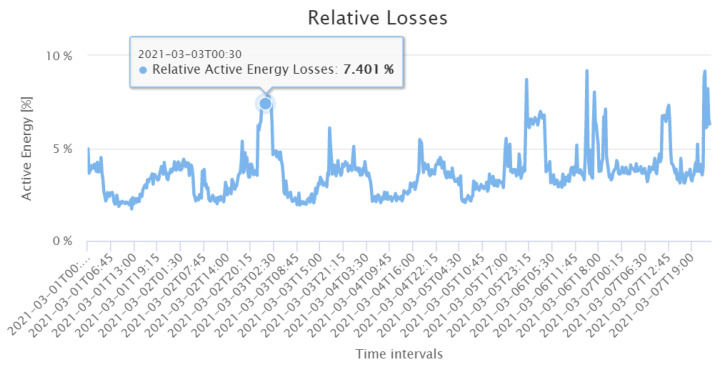
Relative energy losses during the first week of March 2021.

In this study, the loss calculation application was deployed to assess the active power losses of the distribution grid under study. As mentioned in Section 3.2, the loss calculation algorithm computes the ohmic losses in the cables from the line currents and cable parameters. In the remainder, we show GUI screenshots of loss calculation application results for one week in March 2021 (Monday to Sunday) as an example. The grid active energy is calculated at the substation transformer with device 35021, as shown in Figure 13. One can clearly identify the weekend by the smaller energy values. The loss calculation application then subtracts the sum of the individual energy data of the Smart Meters from the absolute value of the transformer energy. The resulting total energy losses are shown in Figure 14. Interestingly, in contrast to the energy values at the transformer, one sees less seasonal fluctuations with respect to the different weekdays. Only fluctuations with respect to the periods at day and night are visible. To derive the relative losses, the total losses are divided by the active energy at the substation. Figure 15 shows that on the analysed period, the relative losses are always below 10%.

## 7. Conclusions

In this paper, a practical approach was followed for deployment of the two observability applications that are identified to be most important to DSOs. A novel architecture was proposed that makes the deployment of the smart applications feasible. At first, a laboratory experimental setup which involves a real-time simulator connected to ICT hardware was utilized for validation of the developed observability applications. The case studies were conducted to validate grid monitoring and loss calculation. The results show that these applications are able to detect voltage quality problems and estimate the line losses to the required accuracy. Field trial results of these applications were also presented in this paper, which proves the feasibility of deployment of the observability applications in real-life distribution grids. Future work involves extending the proposed approach for other smart grid applications, such as outage management and automatic voltage regulation, as well as testing the sensitivity of the observability applications with respect to topology or measurement errors [36]. 

## Figures and Tables

**Table 1:** Hardware/Software Platforms and tools used.

Hardware/Software Platform	Tool/Technology
Opal-RT	RT-Lab/ePHASOR + Matlab/ Simulink
HeadEnds	WebSocket Server
ICT Gateway	Java 1.8 + Jetty
Data Access API	Java Persistence API
Grid Topology Headend	Java 1.8
Grid Observability Apps	Java 1.8
VMS-AMI	Java 1.8
VSS-INV	Java 1.8
GUI	Angular
PCs	Standard Industrial PCs

**Table 2:** Summary of Loss Calculation.

	Energy [kWh]		Error [%]
	True Values from OPAL Data	LC Application with OGM	
Total energy throughput at the substation transformer	172.8	172.7	0.05
Total energy loss (technical loss)	2.91	2.81	3.43

## Abbreviations

The following abbreviations are used in this manuscript:

AMI	Advanced Metering Infrastructure
CIM	Common Information Model
DSO	Distribution system operator
DSSE	Distribution system state estimation
EM	Electrical Measurements
GIS	Geographical Information System
GMON	Grid Monitoring application
GUI	Graphical User Interface
HIL	Hardware in the loop
ICT	Information and communication technologies
INV	Inverter
ICGW	ICT Gateway
LC	Loss Calculation application
LV	Low Voltage
LAN	Local Access Network
MV	Medium Voltage
MAE	Mean Absolute Error
OGM	Observability Grid Model
PAE	Percentage Absolute Error
PMU	Phasor Measurement Unit
PV	Photo Voltaic
PQ	Active and Reactive Power
RES	Renewable Energy Sources
RTU	Remote Terminal Unit
VMS	Virtual Measurement Subsystem
VMS-AMI	VMS for AMI
VSS	Virtual Subsystem
VSS-INV	VSS for PV grid Inverters
